# Brain-derived neurotrophic factor in diabetes mellitus: A systematic review and meta-analysis

**DOI:** 10.1371/journal.pone.0268816

**Published:** 2023-02-14

**Authors:** Fatemeh Moosaie, Soheil Mohammadi, Amene Saghazadeh, Fatemeh Dehghani Firouzabadi, Nima Rezaei

**Affiliations:** 1 School of Medicine, Tehran University of Medical Sciences, Tehran, Iran; 2 Systematic Review and Meta-Analysis Expert Group (SRMEG), Universal Scientific Education and Research Network (USERN), Tehran, Iran; 3 NeuroImaging Network (NIN), Universal Scientific Education and Research Network (USERN), Tehran University of Medical Sciences, Tehran, Iran; 4 Research Center for Immunodeficiencies, Children’s Medical Center, Tehran University of Medical Sciences, Tehran, Iran; 5 MetaCognition Interest Group (MCIG), Universal Scientific Education and Research Network (USERN), Tehran, Iran; 6 Endocrinology and Metabolism Research Center (EMRC), Vali-Asr Hospital, Tehran University of Medical Sciences, Tehran, Iran; 7 Department of Immunology, School of Medicine, Tehran University of Medical Sciences, Tehran, Iran; Chiba Daigaku, JAPAN

## Abstract

**Background:**

Brain-derived neurotrophic factor (BDNF) is a neurotrophic factor expressed in several tissues, including the brain, gut, and pancreas. Activation of the BDNF/TrkB/CREB reduces hepatic gluconeogenesis, induces hepatic insulin signal transduction, and protects against pancreatic beta-cell loss in diabetes mellitus (DM). Several studies have investigated the possible association between BDNF and DM and its complications, but the results have been conflicting.

**Aim:**

In the present study, we aimed at systematically reviewing the literature on the serum and plasma levels of BDNF in DM and its subgroups such as T2DM, DM patients with depression, and patients with retinopathy.

**Methods:**

A comprehensive search was conducted in PubMed, Scopus, and Web of Science. We identified 28 eligible studies and calculated the standardized mean difference (SMD) of outcomes as an effect measure.

**Results:**

The meta-analysis included 2734 patients with DM and 6004 controls. Serum BDNF levels were significantly lower in patients with DM vs. controls (SMD = -1.00, P<0.001). Plasma BDNF levels were not different in patients with DM compared with controls. When conducting subgroup analysis, serum BDNF levels were lower among patients with T2DM (SMD = -1.26, P<0.001), DM and depression (SMD = -1.69, P<0.001), and patients with diabetic retinopathy (DR) vs. controls (SMD = -1.03, P = 0.01).

**Conclusions:**

Serum BDNF levels were lower in patients with DM, T2DM, DM with depression, and DM and DR than the controls. Our findings are in line with the hypothesis that decreased BDNF levels might impair glucose metabolism and contribute to the pathogenesis of DM and its complications.

## 1. Introduction

Brain-derived neurotrophic factor (BDNF) is a neurotrophic factor that acts as a neurotransmitter modulator contributing in survival and plasticity of neurons as well as maintenance and growth of β cells of the pancreas [1,2].

Diabetes mellitus (DM) is a major concern in healthcare worldwide, with high morbidity and mortality. DM is characterized by hyperglycemia which is a result of a metabolic defect; including distorted insulin secretion, action or both. Type 1 diabetes mellitus (T1DM), which accounts for only 5–10% of those with DM, develops due to destruction of β-cells; progression of the disease eventually leads to absolute insulin deficiency. Type 2 diabetes mellitus (T2DM), which accounts for 90–95% of those with DM, results from a combination of insulin resistance and relative insulin deficiency. Gestational diabetes mellitus (GDM) is defined as glucose intolerance which develops for the first time during pregnancy [3].

BDNF is involved in β cell survival by binding to tyrosine kinase B (TrkB) -its high-affinity receptor-and activating IRS1/2, PI3K, Akt pathway which lead to expression of genes encoding proteins responsible for cell survival. Downstream signaling cascade of BDNF is similar to insulin-like growth factor-1, including p-CAMK and MAPK; the pathway is involved in expression of pro-survival genes. Then, BDNF prevents exhaustion of β cells and regulates glucose and energy metabolism [2,4,5].

Recent evidence suggests that BDNF is associated with cognitive dysfunction and is decreased in various neurologic and psychiatric disorders like depression, Alzheimer’s disease, and Parkinson’s disease [6–8]. Knowing that patient with DM are susceptible to cognitive impairment and neuropsychiatric disorders [9], one could suggest that BDNF levels might play a role in DM-mediated cognitive decline. Also, regarding the role of BDNF in metabolism [10], it might be speculated that circulating BDNF levels might alter as a result of the disturbance in glucose-associated pathways in DM patients.

Several studies have been conducted on the association between BDNF and DM and its complications. However, the results have been conflicting. Some of the studies have shown significantly decreased levels of BDNF [5,11–25] and some others increased levels in patients with DM [26–29]. Recently, Davarpanah and colleagues performed a meta-analysis on circulating BDNF levels in DM subjects compared with healthy individuals [30]. The results of this meta-analysis showed that serum BDNF levels are significantly lower in DM subjects compared with healthy individuals. However, the authors of this study only included adults with type 2 DM. Moreover, the authors of this study did not consider the neuropsychiatric complication of diabetes such as depression as a probable moderator of BDNF levels while recent evidence suggest that these complications might alter serum BDNF levels. Also, the authors used weighted mean difference as a measure of effect size while due to a high degree of between study heterogeneity in measurement of BDNF levels standardized mean difference seems to be a better measurement scale. In the present study, we aimed at systematically reviewing the literature on the serum and plasma levels of BDNF in DM and its subgroups such as T2DM, DM patients with depression, and patients with diabetic retinopathy (DR). We also integrated previous findings using meta-analysis.

## 2. Methods

### 2.1. Protocol and registration

We employed the Preferred Reporting Items for Systematic Reviews and Meta-analyses (PRISMA) guideline to develop the study protocol and articulate the aims of the review, methods for search, data collections and analysis. We also determined the inclusion and exclusion criteria of the participants, studies, intervention and outcome (PICO questions. The study protocol was submitted at the PROSPERO [31].

### 2.2. Search strategy

The primary inclusion criteria were English original articles that measured BDNF concentrations in serum, plasma, umbilical cord, aqueous or vitreous humor levels of BDNF in patients with DM. We assigned no limitation for the date or status (i.e. online first or published) of the publication. We searched PubMed, Scopus, and Web of Science for studies matching the PICO question using a combination of keywords provided in Supplementary 1. The last search took place in October 2020. Moreover, the reference lists were screened for remaining relevant studies, corresponding authors were contacted. The was included in the review in the case of receiving response.

### 2.3. Selection criteria

Inclusion criteria was as follows: Original articles (i.e. case-control and/or cohort) on human subjects; with participants with DM vs. controls; which their diagnosis of DM was established by an endocrinologist based on American diabetes association (ADA) criteria [3]; and had measured levels of BDNF in samples of the serum, plasma, umbilical cord, or vitreous humor in patients with DM; with the use of techniques such as immunoassay, enzyme-linked immunosorbent assay (ELISA) and western blot; with reported data on the total number of participants and mean and standard deviation (SD) of BDNF levels in both groups cases and controls.

We excluded books, review articles, personal opinions, letters, conference abstracts and grey literature; studies with no control group; studies on animal subjects; studies that measured expression of BDNF in the tissues; and studies that were designed in-vitro or on cell cultures. Language, publication time, participants’ group of age, and type of DM were not limited.

### 2.4. Data collection

Eligible studies were evaluated by two experts independently and the following data was extracted from each included publication: author; date of publications; country of origin; study design; definition, inclusion, exclusion criteria and the number of case and controls; variables matched, the proportion of male and obesity, mean age, duration of disease, fasting blood glucose (FBS), HbA1c, body mass index (BMI), mean (SD) of BDNF levels among cases and controls; the proportion of retinopathy, proliferative retinopathy, neuropathy and nephropathy among cases; type, time and assay used for sampling. Any conflicts in data extraction were discussed or consulted by a third expert and resolved.

### 2.5. Quality assessment

The Newcastle–Ottawa Scale (NOS) was employed to assess the quality of included observational studies in terms of three main aspects; sample selection, comparability of cases and controls, and exposure [32]. Calculated scores range from 0 to 9. Studies with scores below 4 are least reliable with highest risk of bias; Scores of 4 to 6 represent moderate risk of bias and quality; Studies scored from 7 to 9 are the most reliable with the lowest risk of bias. Moreover, the studies were evaluated in terms of methodology by two experts, independently; any conflict of opinion was discussed or referred to third expert and resolved.

### 2.6. Statistical analysis

STATA version 16 for Windows (Stata Corp, College Station, Texas) was utilized for the meta-analysis. At least three studies with five or more participants in each group were required to synthesize the data on outcomes. The heterogeneity of studies was measured using I^2^ or Q test. According to the handbook of Cochrane, an I^2^ level of below 40% is considered as a low level of heterogeneity. As a result, we interchangeably used fixed-effects model or random-effects model based on heterogeneity of studies. A fixed model was employed, if the heterogeneity of studies was below 40% and a random effect model in case of heterogeneity above 40%. Effect measures were calculated for the standardized mean difference (SMD) of outcomes. Also, based on the heterogeneity of studies, either meta-regression analysis or subgroup analysis was performed for potential moderators. Moreover, funnel plot asymmetry and the Eggers test were used to assess publication bias. In case of significant publication bias, the adjustment was done for the effect size using the trim and fill method. A P-value less than 0.05 was considered statistically significant.

## 3. Results

### 3.1. Study selection

Database search resulted in 859 records. Seven hundred forty-one studies were primarily excluded based on title and abstract. The remaining 118 articles were fully studied and the articles that met the inclusion criteria were extracted, leaving us with 28 studies. The remaining articles were carefully evaluated. The corresponding authors were contacted, in case that the mean (SD) of BDNF levels were not reported.

**Fig 1:**
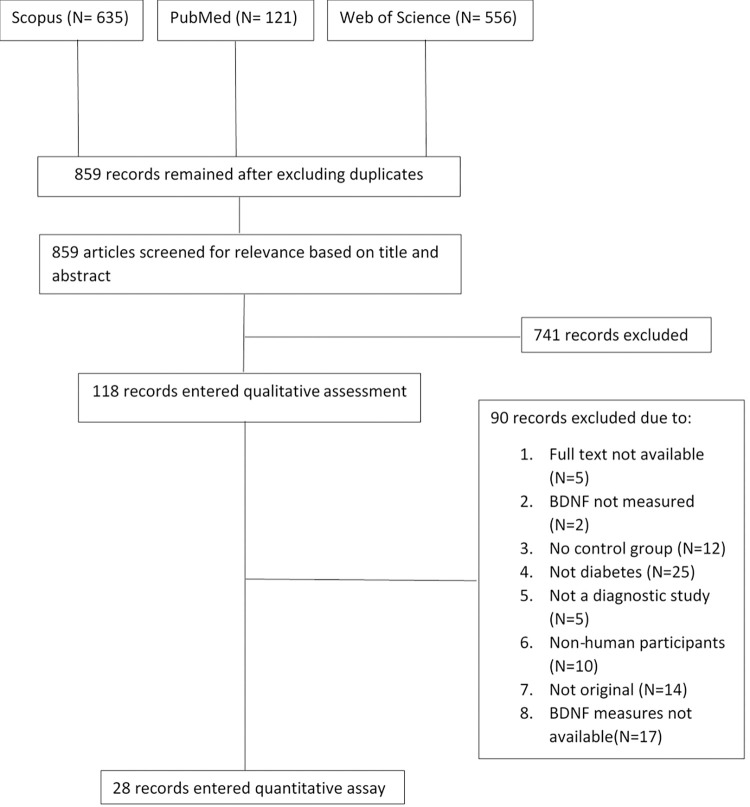
Search method and study selection.

Fig 1 presents the steps of the study selection in more detail.

### 3.2. Characteristics of the included studies

Included studies dated from 2006 to 2019. Sample sizes of the studies varied from 18 to 1850. The mean age of the participants ranged from 8.74 to 66. The majority of the studies had a case-control design (n = 25), and the rest were cohort studies. Thirty studies measured serum and plasma BDNF levels in patients with DM and controls, of which twenty were T2DM. Three studies measured BDNF levels in patients with DM and depression vs. controls.

Table 1 presents the detailed characteristics of the included studies.

**Table 1:** Characteristics of the included studies.

First author (year)	country	No of DM	No of HC	Age in DM	Age in HC	Male (%) in DM	Male (%) in HC	BDNF sample	BDNF measurement	Mean BDNF(SD) (ng/ml) in DM	Mean BDNF (SD) (ng/ml) in HC
Zhen 2019	China	230	248	55.15	55.21	41.73	41.93	Serum	ELISA	7.51(0.18)	11.54(0.18)
Spartano 2019 [38]	USA	179	1551	64	60	61	54	Serum	ELISA	24.343(8.335)	23.881(7.124)
Nanri 2019 [28]	Japan	54	1796	-	-	3.1	90.5	Serum	ELISA	32.1(38.4)	30.1(4.94)
Sun, Z 2018 [19]	China	151	396	66	-	44.4	-	Serum	ELISA	23.5(8.37)	28.1(5.93)
Sun, Q 2018 [20]	China	83	110	56.5	57.3	97.6	74.6	Serum	ELISA	2.1268(0.1711)	2.2467(0.3315)
Zhen 2017	China	311	346	54.93	53.43	43.7	39.9	Serum	ELISA	7.73(2.95)	11.56(2.67)
zhange 2017	China	15	15	14.26	17.87	0	0	Serum	ELISA	14.26(11.4)	17.87(10.18)
Guo 2017 [14]	China	291	212	55	-	55.3	54.7	Serum	ELISA	17.8 (5.93)	24.6 (7.48)
Wei 2016	China	92	81	61	61	54.3	52.1	Serum	ELISA	1.32(0.36)	1.77(0.77)
Ortiz 2016 [51]	Mexico	19	40	50.57	42	100	100	Serum	ELISA	31.55(10.24)	39.36(8.9)
Liu 2016 [5]	China	28	105	45	46	0	38.8	Serum	ELISA	23.4(9.05)	24.71(6.93)
Li 2015	China	137	200	60	60	51	-	Serum	ELISA	10.8(3.63)	24.6(7.85)
Kavirasan 2015	India	27	27	51	44	37.03	96.2	Serum	ELISA	0.06365(0.03007)	0.07345(0.0323)
Wang 2014	China	20	30	56.75	57	-	-	Serum	-	22.25(5.69)	33.08(14.47)
Passaro 2014	Italy	27	94	-	-	-	-	Plasma	Immunoassay	0.2649(0.136)	0.301(0.1307)
Marchelek 2014	Poland	11	20	58.1	56.4	63.6	55	Plasma	ELISA	2.5531(2.0704)	4.3541(3.3915)
Qiu 2014	China	56	60	-	-	-	-	Serum	ELISA	6.88(3.1)	9.97(2.59)
Lee 2014 [37]	Korea	7	11	15.5	16.4	28.57	100	Serum	ELISA	25.1361(3.81416)	21.1783(4.30614)
He 2014	China	37	37	54.9	55.4	67.56	70.27	Serum	-	22.04(6.79)	28.53(16.14)
Boyuk 2014 [26]	Turkey	88	33	60.03	58.2	43.2	51.5	Serum	ELISA	0.20681(0.10732)	0.13084(0.05981)
Zhou 2013 [25]	China	232	70	53.1	55.2	55.6	48.57	Serum	ELISA	0.07984(0.00515)	0.08529(0.00327)
Zhen 2013 [24]	China	208	212	51.4	50.8	41.34	43.39	Serum	ELISA	8(2.9)	11.9(2.6)
Ola 2013 [17]	Saudi Arabia	22	19	48.7	51.3			Serum	ELISA	21.8(4.9)	25.5(8.5)
Kim 2013 [34]	Korea	8	30	51.4	47	100	100	Plasma	Immunoassay	1.3638(1.1646)	1.6288(1.4274)
Civelek 2013 [27]	Turkey	90	35	53.5	57.1	80	75	Serum	ELISA	1.6268(0.1708)	0.6905(0.2508)
Abu El-Asr 2013 [11]	Saudi Arabia	46	34	53.9	47.8	76.08	67.64	Serum	ELISA	10.2(7.7)	16.7(10.3)
Fujinami 2008 [13]	Japan	112	80	57.8	57.6	47.5	47.32	Serum	ELISA	15.5(5.2)	20(7.3)
Suwa 2006 [29]	Japan	24	7	51	47.6	0	0	Serum	ELISA	40.6(9.9)	30.6(7.2)

BDNF, brain-derived neurotrophic factor; DM, diabetes mellitus; HC, healthy controls; SD, standard deviation; ELISA, enzyme-linked immunoassay.

### 3.3. Quality assessment of the included studies

Evaluation of the quality of studies was based on three aspects; participant selection, comparability of cases and controls, and exposure determination. Twenty-two studies had sufficient selection of the cases and controls; cases and controls of fifteen studies were matched for age and sex, and only one for BMI additionally. All studies had sufficient quality of determining exposure.

Details of the quality assessment of each if the included studies are presented in Supplementary 2.

### 3.4. BDNF levels in DM patients vs. control

**Fig 2:**
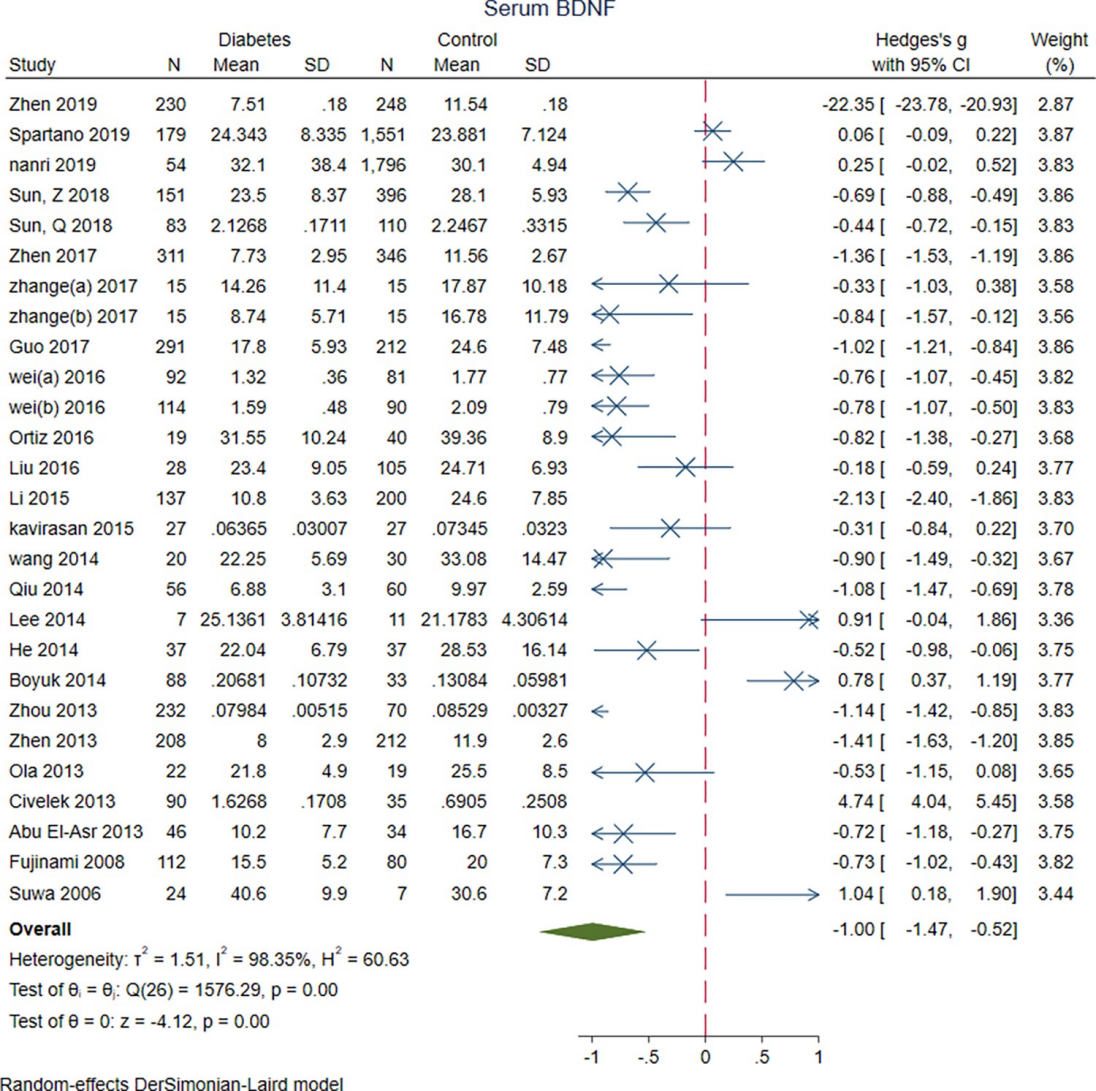
A meta-analysis of serum BDNF levels in patients with DM vs. controls.

**Fig 3:**
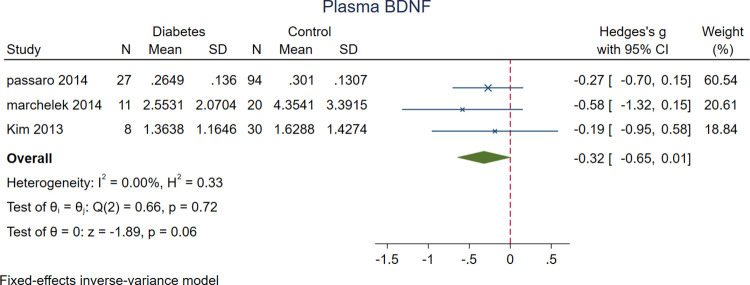
A meta-analysis of plasma BDNF levels in patients with DM vs. controls.

Meta-analysis of twenty-five studies revealed significantly lower serum BDNF levels in patients with DM (n = 2688) compared to the controls (n = 5860) (SMD = -1.00 [-1.47, -0.52], P<0.001). Plasma BDNF levels was lower in patients with DM (n = 46) vs. controls (n = 144) in three of the studies. (SMD = -0.32 [-0.65, 0.01], P = 0.06); however, the differences were not statistically significant (Figs 2 and 3).

### 3.5. Serum BDNF levels in T2DM patients vs. control

**Fig 4:**
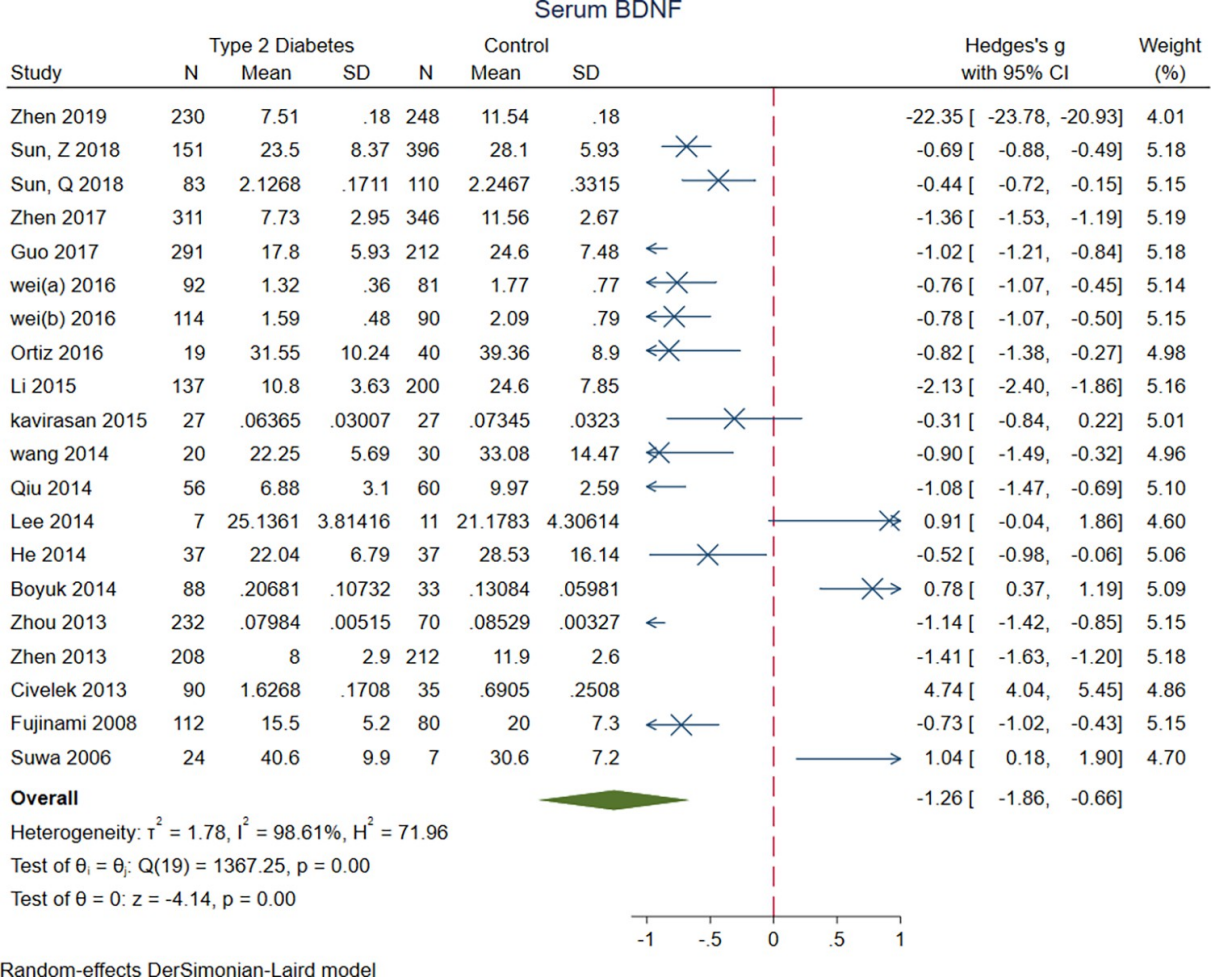
A meta-analysis of serum BDNF levels in patients with T2DM vs. controls.

Meta-analysis of eighteen studies revealed significantly lower serum BDNF levels in T2DM patients (n = 2329) compared to the controls (n = 2325) (SMD = -1.26 [-1.86, -0.66], P<0.001) (Fig 4).

### 3.6. Serum BDNF levels in DM patients with depression vs. control

**Fig 5:**
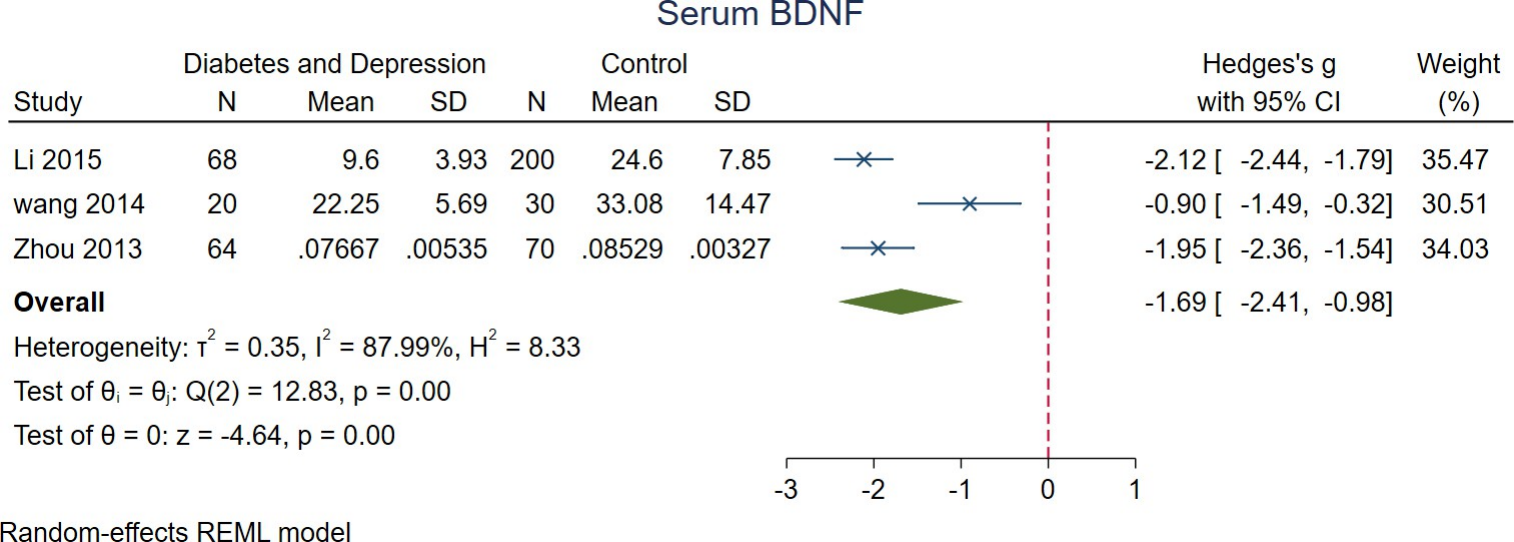
A meta-analysis of serum BDNF levels in patients with DM and depression vs. controls.

Meta-analysis of three studies showed significantly lower levels of serum BDNF in DM patients with depression (n = 152) compared to the controls (n = 300) (SMD = -1.69 [-2.41, -0.98], P<0.001) (Fig 5).

### 3.7. Serum BDNF levels in DM patients with DR vs. control

**Fig 6:**
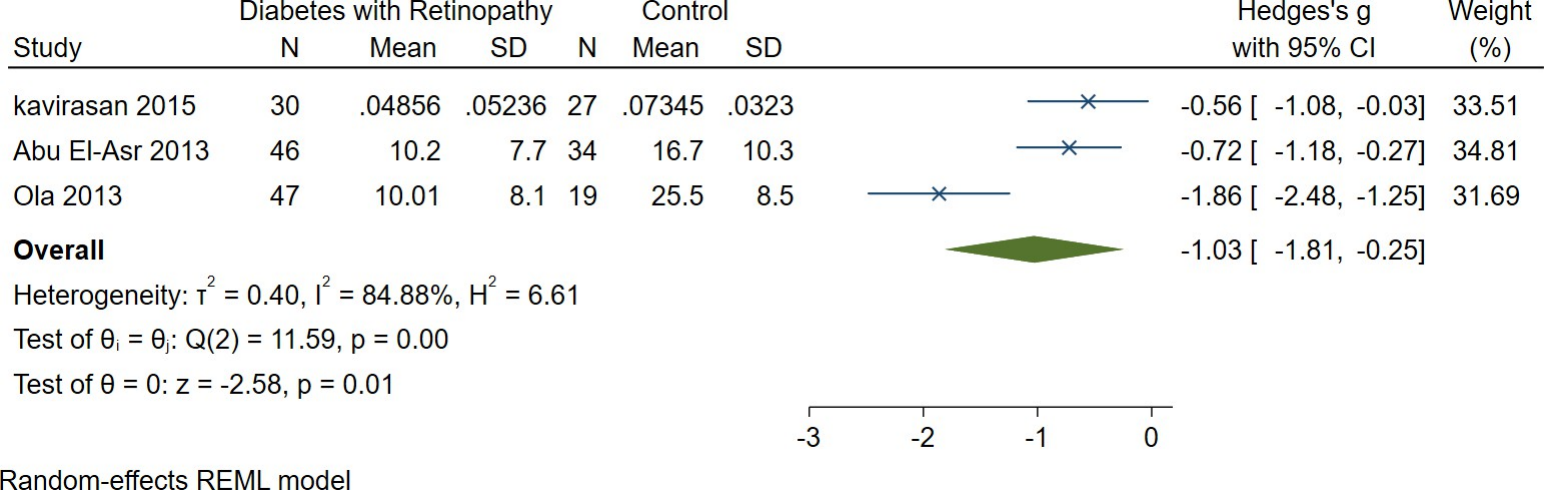
A meta-analysis of serum BDNF levels in patients with DM and retinopathy vs. controls.

Meta-analysis of three studies demonstrated significantly lower levels of serum BDNF in patients with DM and DR (n = 123) compared to the controls (n = 80) (SMD = -1.03 [-1.81, -0.25], P = 0.01) (Fig 6).

### 3.8. Cumulative meta-analysis: Sample size, age, duration of DM, FBS and HbA1c

**Fig 7:**
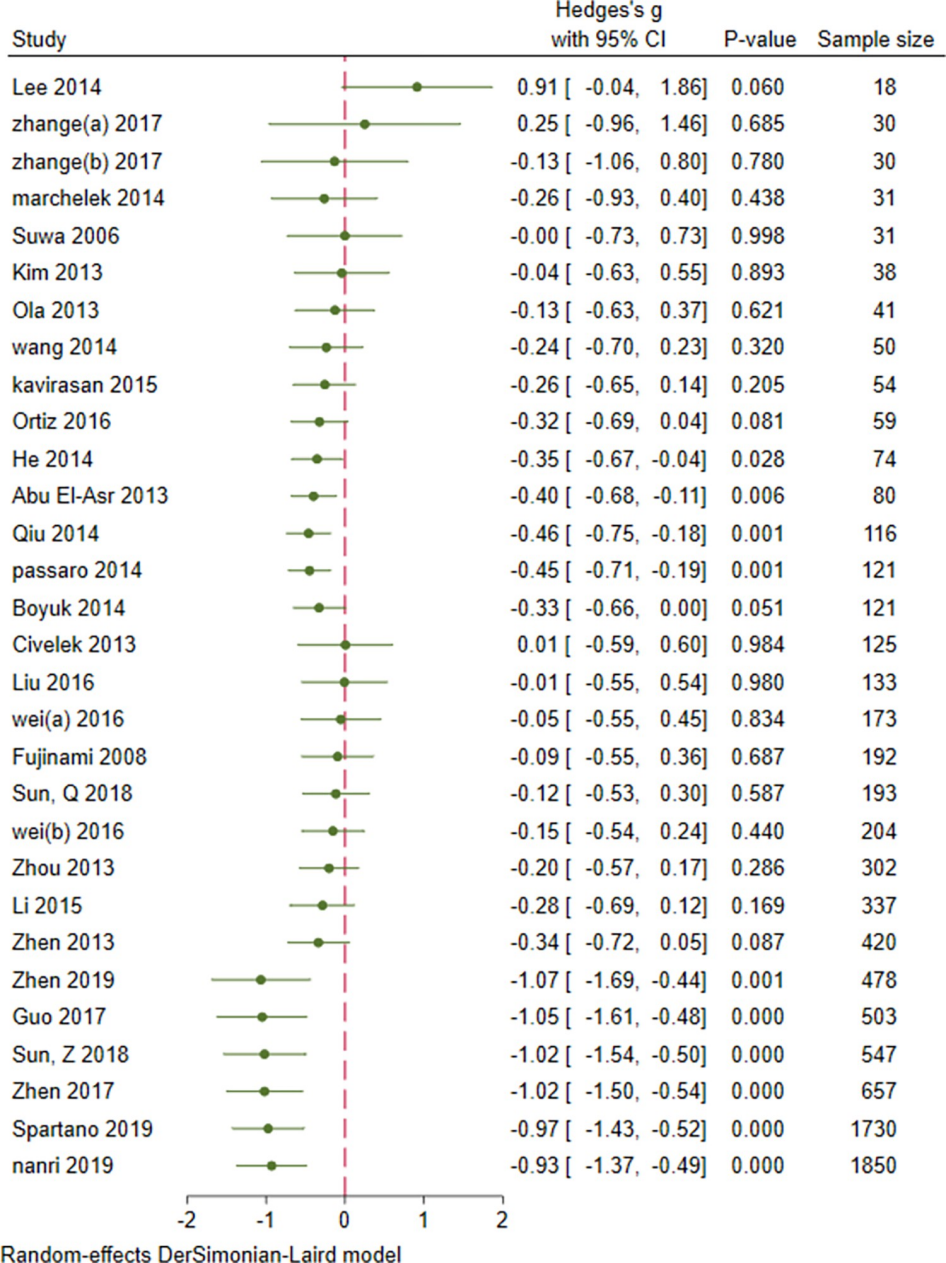
Cumulative meta-analysis of BDNF levels in patients with DM by sample size.

**Fig 8:**
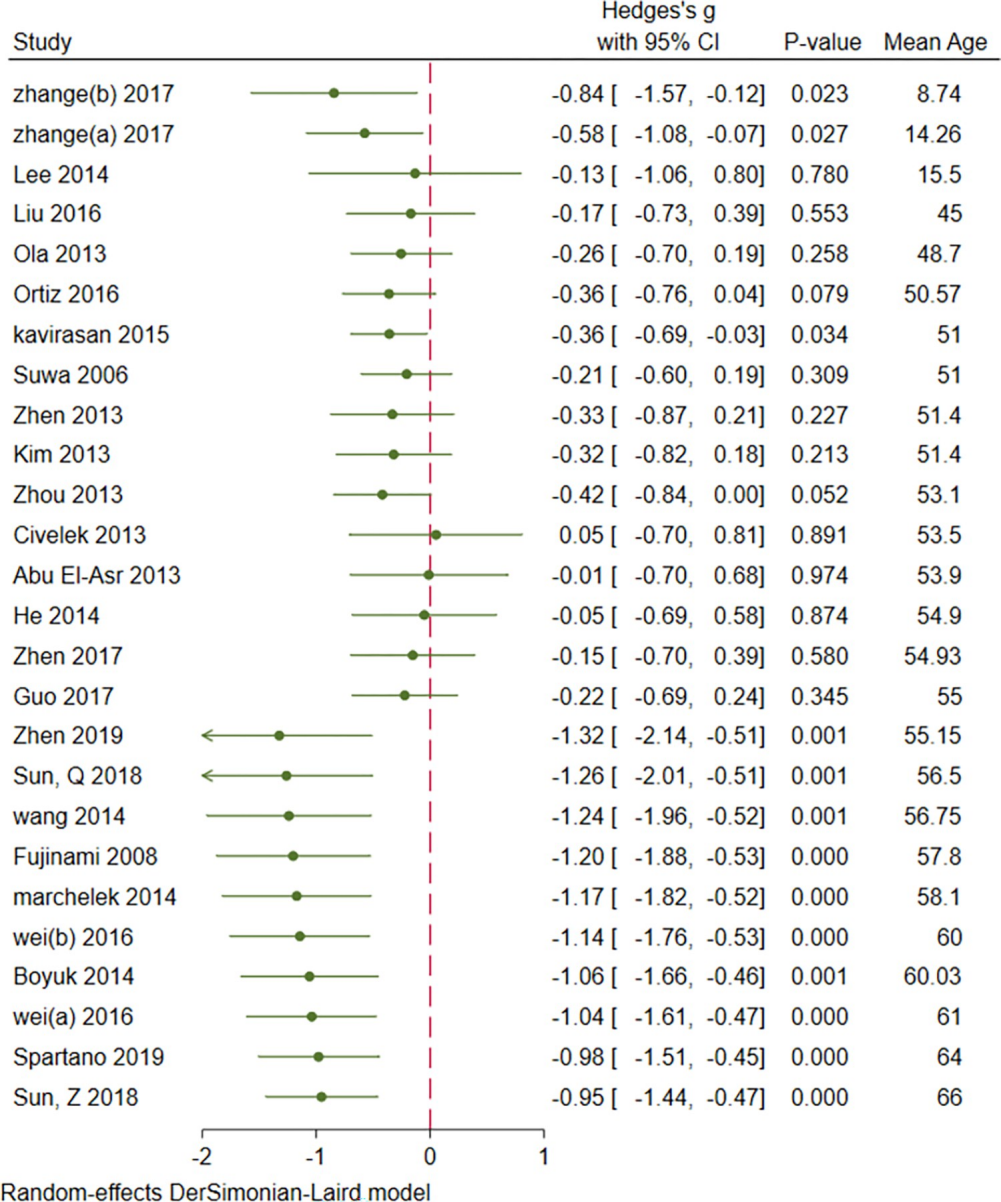
Cumulative meta-analysis of BDNF levels in patients with DM by the mean age of cases.

**Fig 9:**
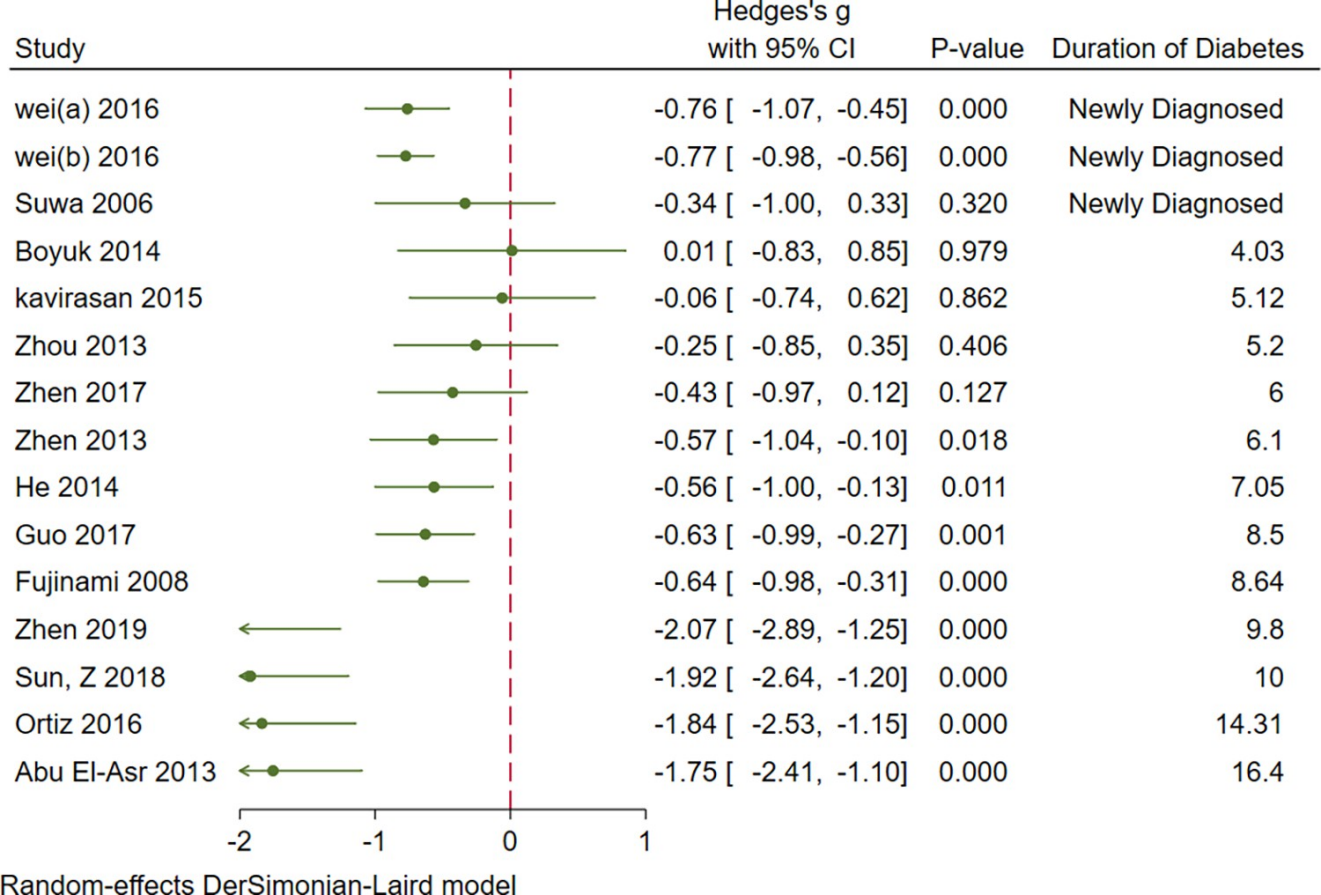
Cumulative meta-analysis of BDNF levels in patients with DM by the duration of the disease.

**Fig 10:**
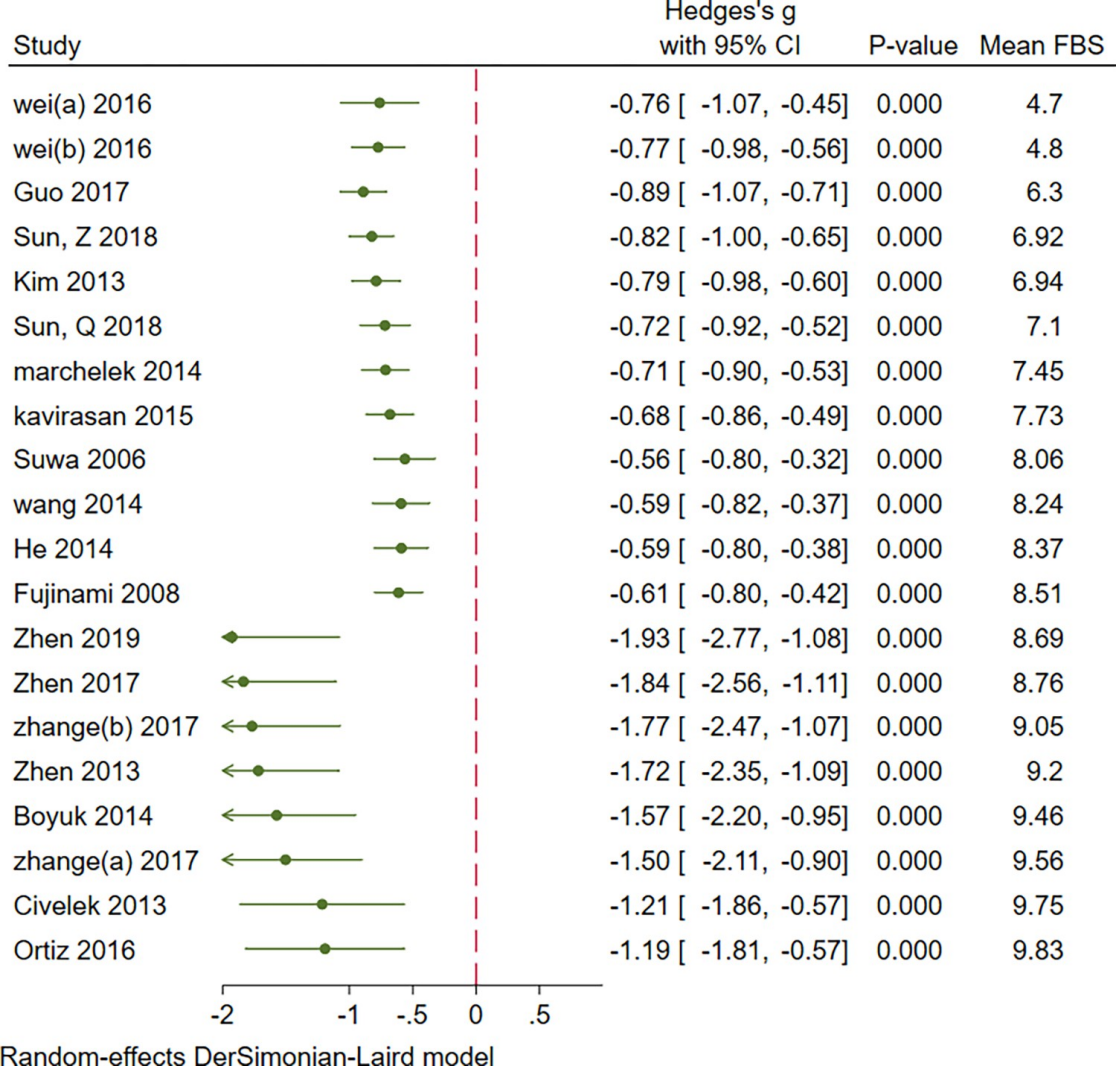
Cumulative meta-analysis of BDNF levels in patients with DM by mean FBS of cases.

**Fig 11:**
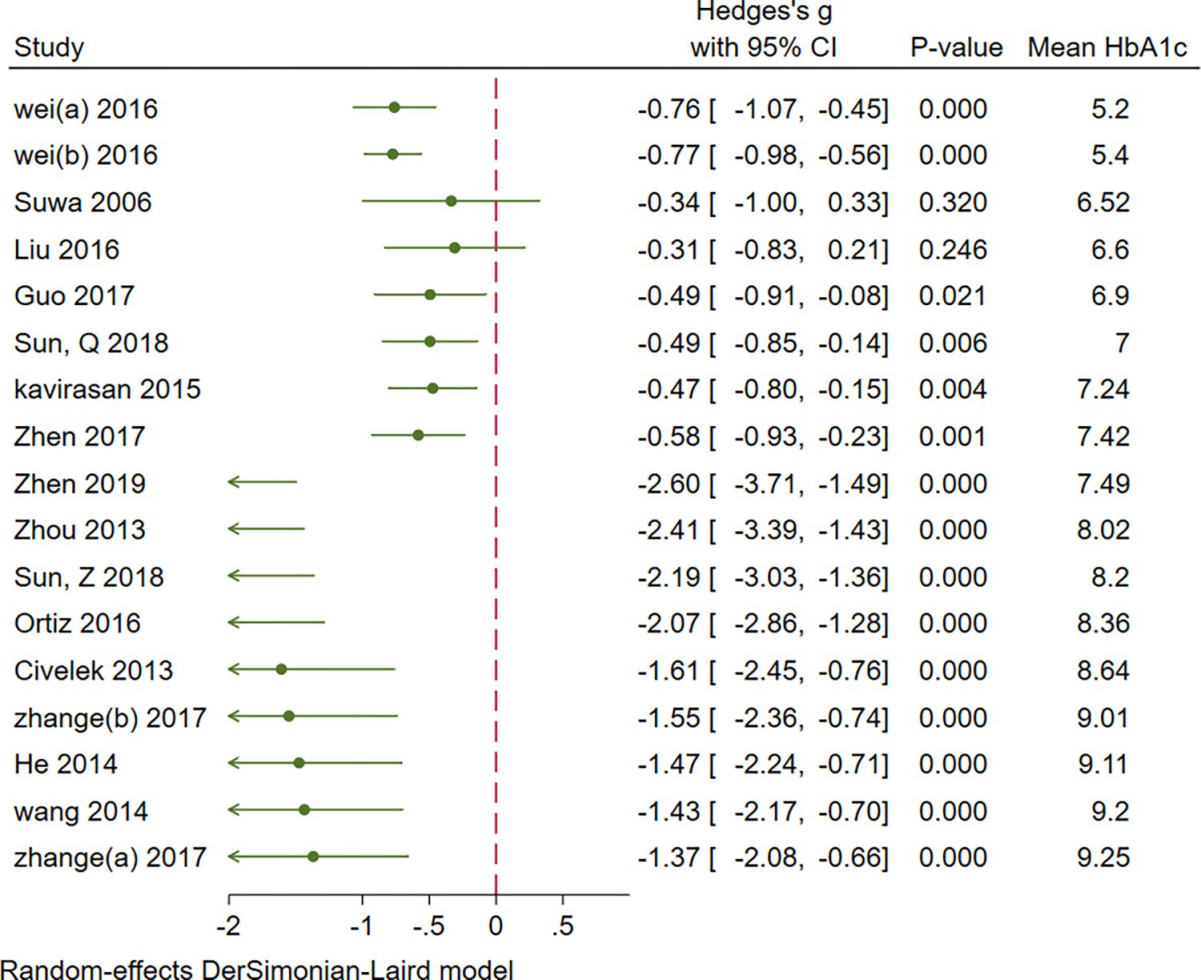
Cumulative meta-analysis of BDNF levels in patients with DM by mean HbA1c of cases.

Cumulative analysis of serum BDNF levels in patients with DM showed an overall decrease in effect size with an increase in sample size, mean age, duration of DM, FBS, and mean HbA1c. Figs 7–11 show a variation in the effect size of BDNF in studies with mean age of above 55 years, duration of diabetes above 10 years, mean FBS above 8.69 mmol/L and Hba1c above 7.49. The effect This shows that the longer the patients are exposed to high glucose levels and the more severe their condition is, the lower the BDNF would be.

### 3.9. Subgroup meta-analysis: Study design, sex, study design, BMI and age and continent

**Fig 12:**
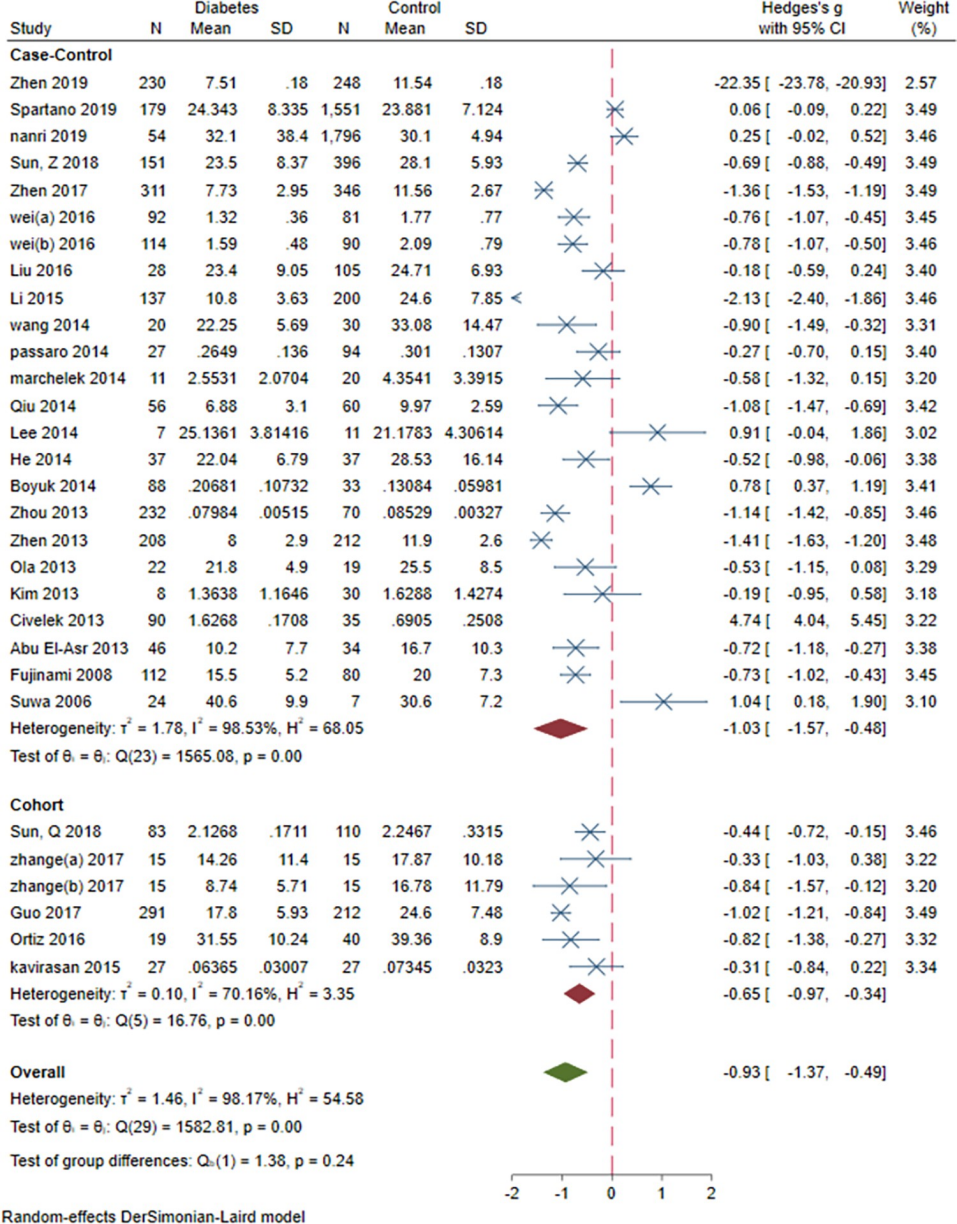
Subgroup meta-analysis of BDNF levels in patients with DM by study design.

**Fig 13:**
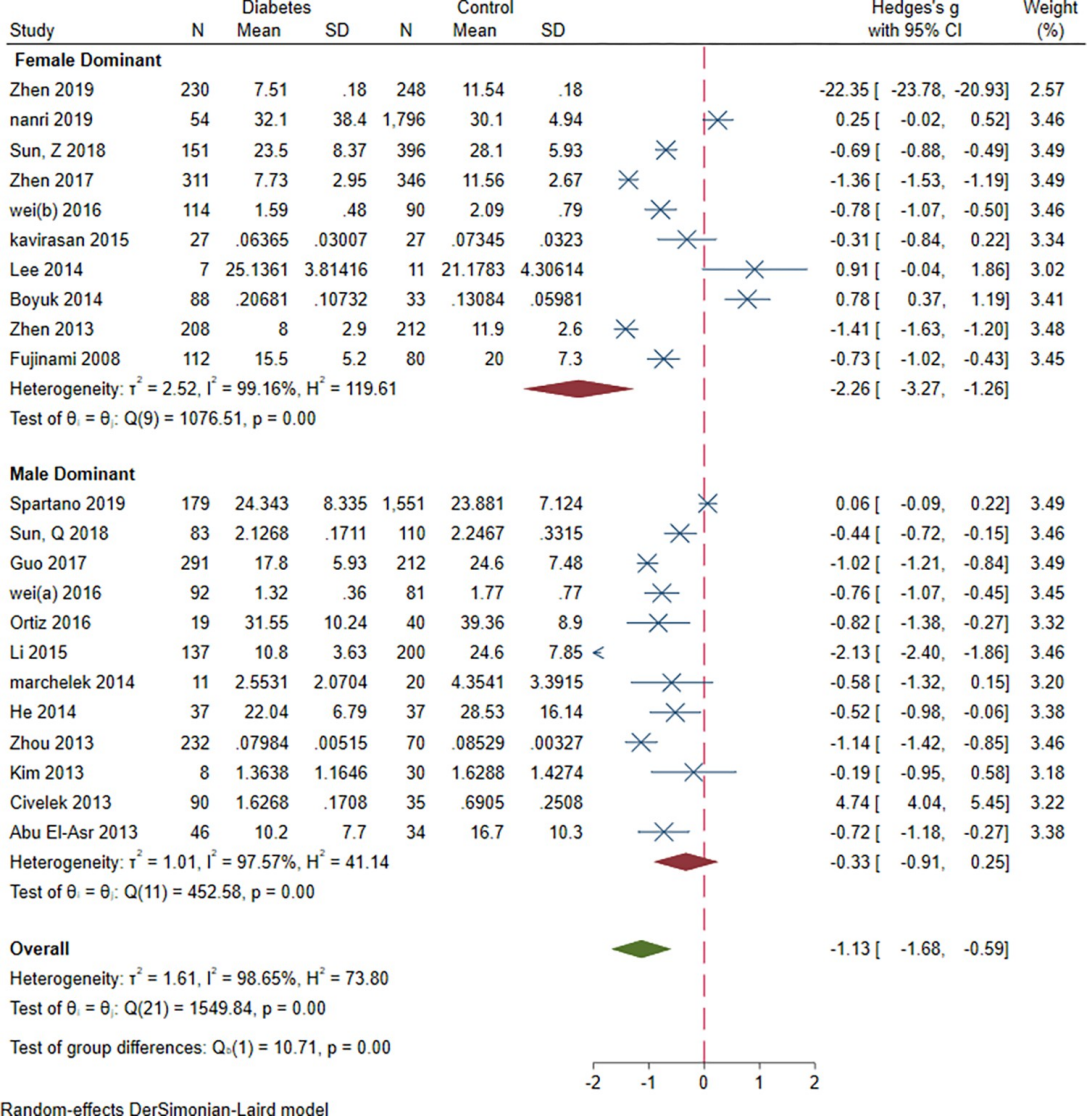
Subgroup meta-analysis of BDNF levels in patients with DM by gender dominance of the study population.

**Fig 14:**
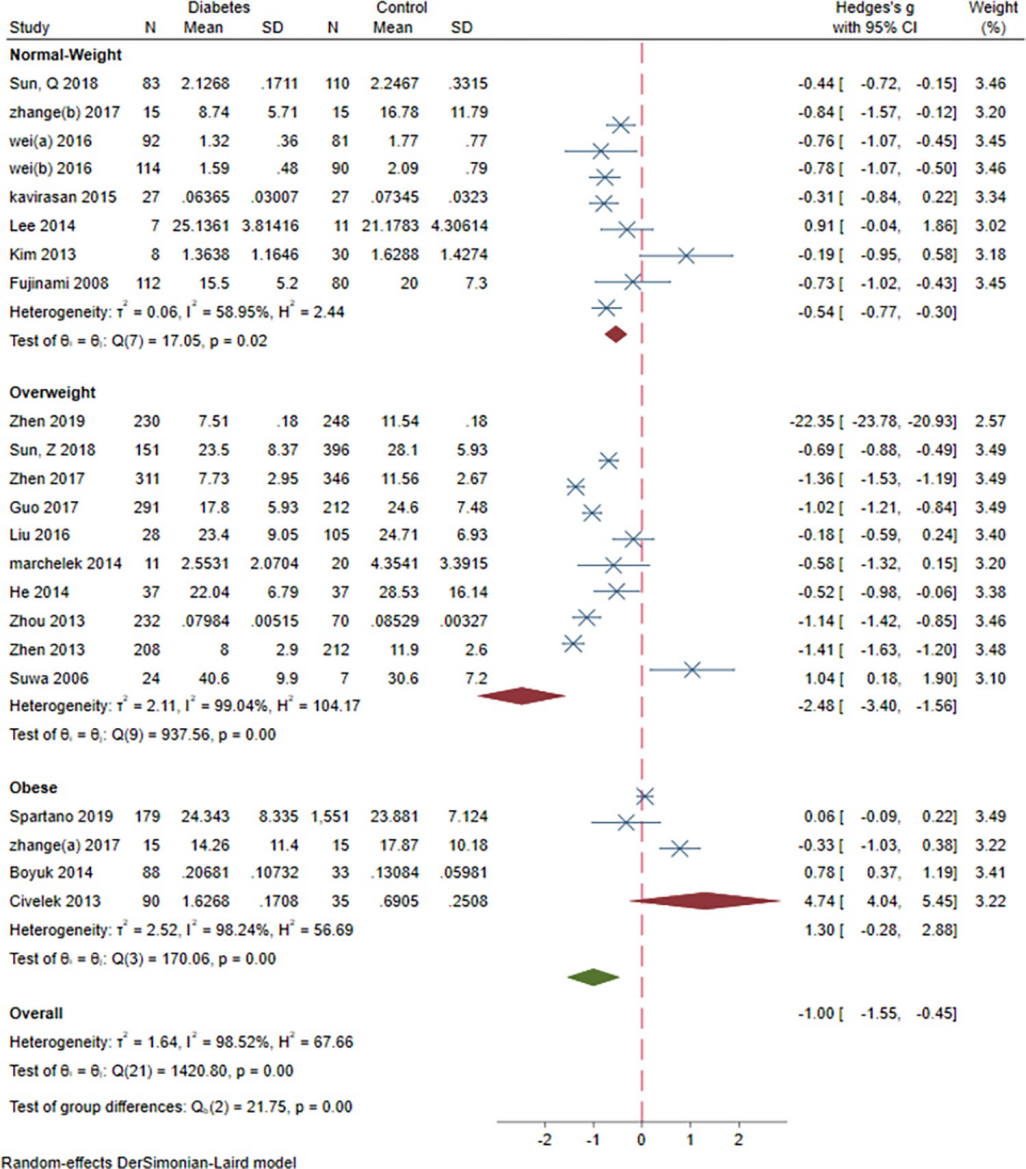
Subgroup meta-analysis of BDNF levels in patients with DM by BMI-based weight status.

**Fig 15:**
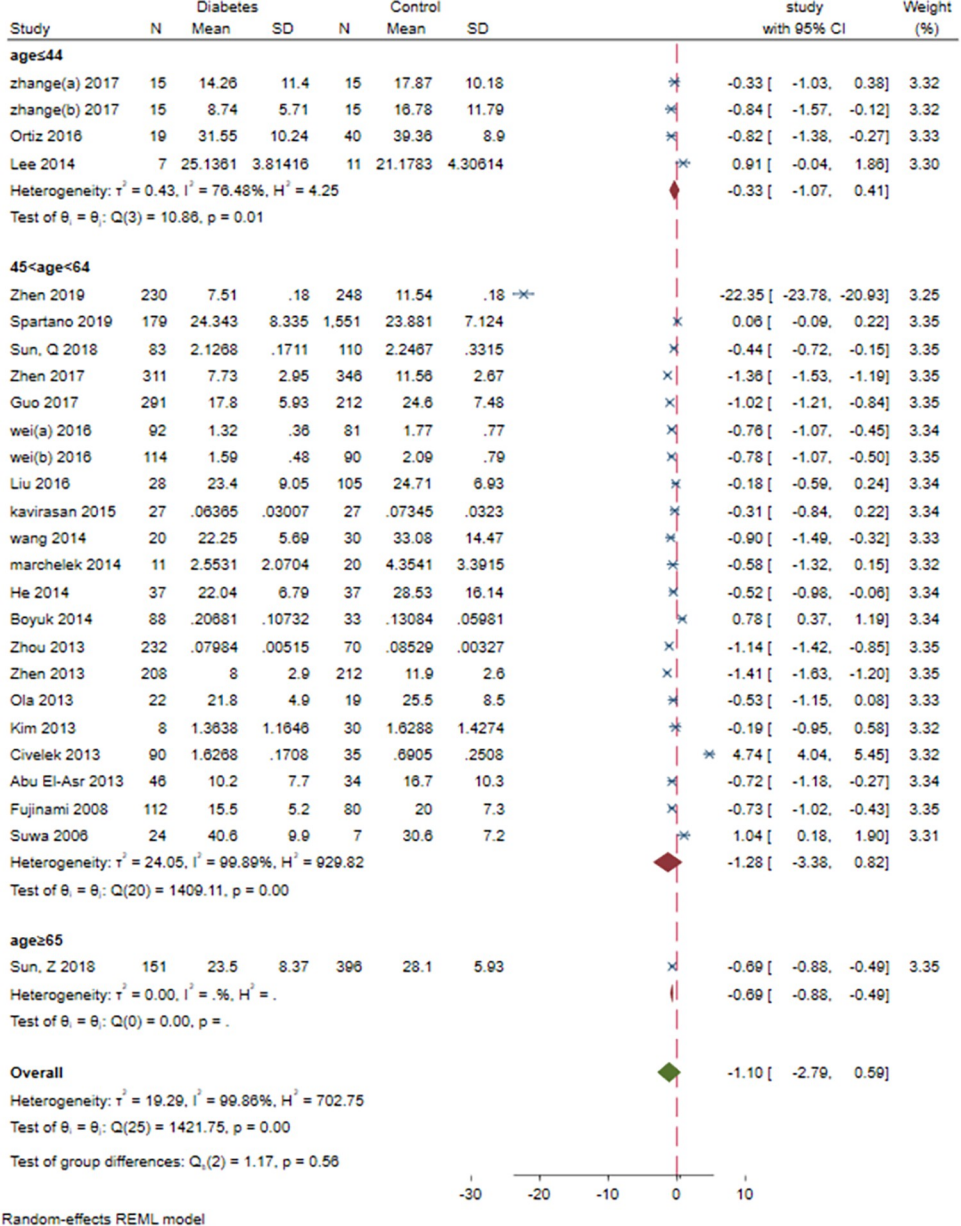
Subgroup meta-analysis of BDNF levels in patients with DM by age.

**Fig 16:**
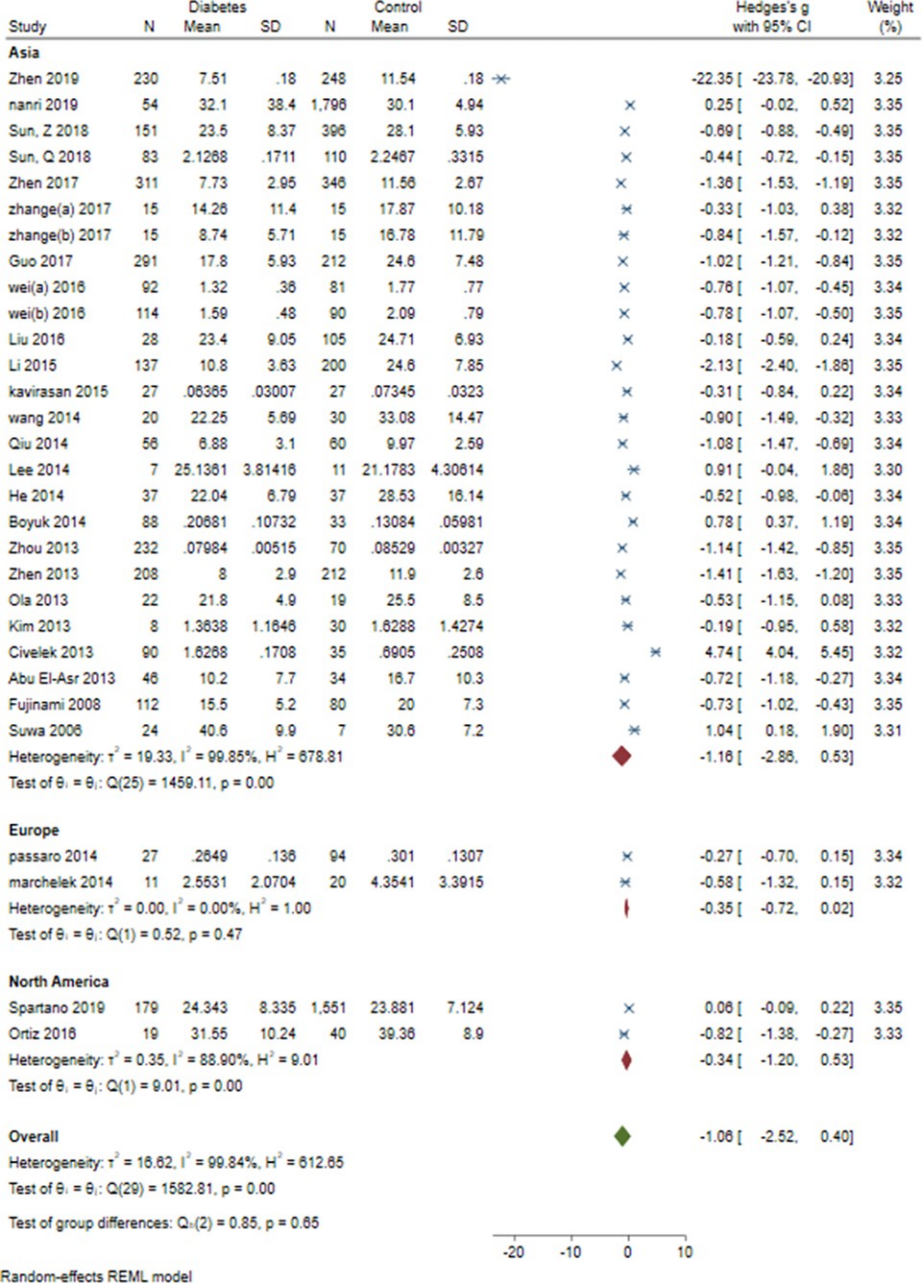
Subgroup meta-analysis of BDNF levels in patients with DM by continent.

Subgroup meta-analysis of serum BDNF levels in DM based on study design revealed a significantly lower levels of serum BDNF in patients with DM compared vs. controls in case-control and cohort studies (SMD = -1.03[-1.57, -0.48], P = 0.00023 and SMD = -0.65[-0.97, -0.34], P = 0.0006, respectively). Subgroup meta-analysis based on sex revealed significantly lower levels of BDNF in DM compared to control in female-dominant studies (SMD = -2.26[-3.27, -1.26], P = 0.00001). Subgroup meta-analysis based on BMI revealed significantly lower levels of BDNF in normal BMI and overweight patients with DM (SMD = -0.54[-0.77, -0.3], P = 0.00001 and SMD = -2.48[-3.4, -1.56], P<0.00001, respectively). Subgroup analysis based on study design (case-control vs. cohort), revealed significantly lower level of BDNF in case-control (SMD = -1.03[-1.57, -0.48], P<0.00001) and cohort (SMD = -0.65[-0.97, -0.34], P<0.00001) studies. Subgroup analysis based on the continent revealed that most of the studies (N = 24) were conducted in Asia (SMD = -1.16[-2.86, 0.53]). Two studies were conducted in Europe (SMD = -0.35[-0.72, 0.02]) and another two in North America (SMD = -0.34[-1.20, 0.53]). There was no significant between-group difference based on study design (P = 0.24), age(P = 0.56) and continent (P = 0.65). However, there was a significant difference based on sex and BMI (both P<0.00001) (Figs 12–16).

### 3.10. Meta-regression analysis

Univariate meta-regression analyses for serum BDNF in patients with DM revealed no significant the between-study difference based on sex, age, publication year, sample size, FBS, HbA1c, and BMI. A significant negative correlation was found between the effect size and duration of DM in 15 observations (slope = -0.55 [-.79, -0.31], R^2^ = 64.40%, P<0.001), when comparing serum BDNF levels between patients with DM and controls. In comparison of serum BDNF levels in T2DM patients versus controls, effect size was significantly and negatively associated with duration of DM in 14 observations (slope = -0.604[-0.88, 0.33], R^2^ = 61.45%, P<0.001).

### 3.11. Publication bias

**Fig 17:**
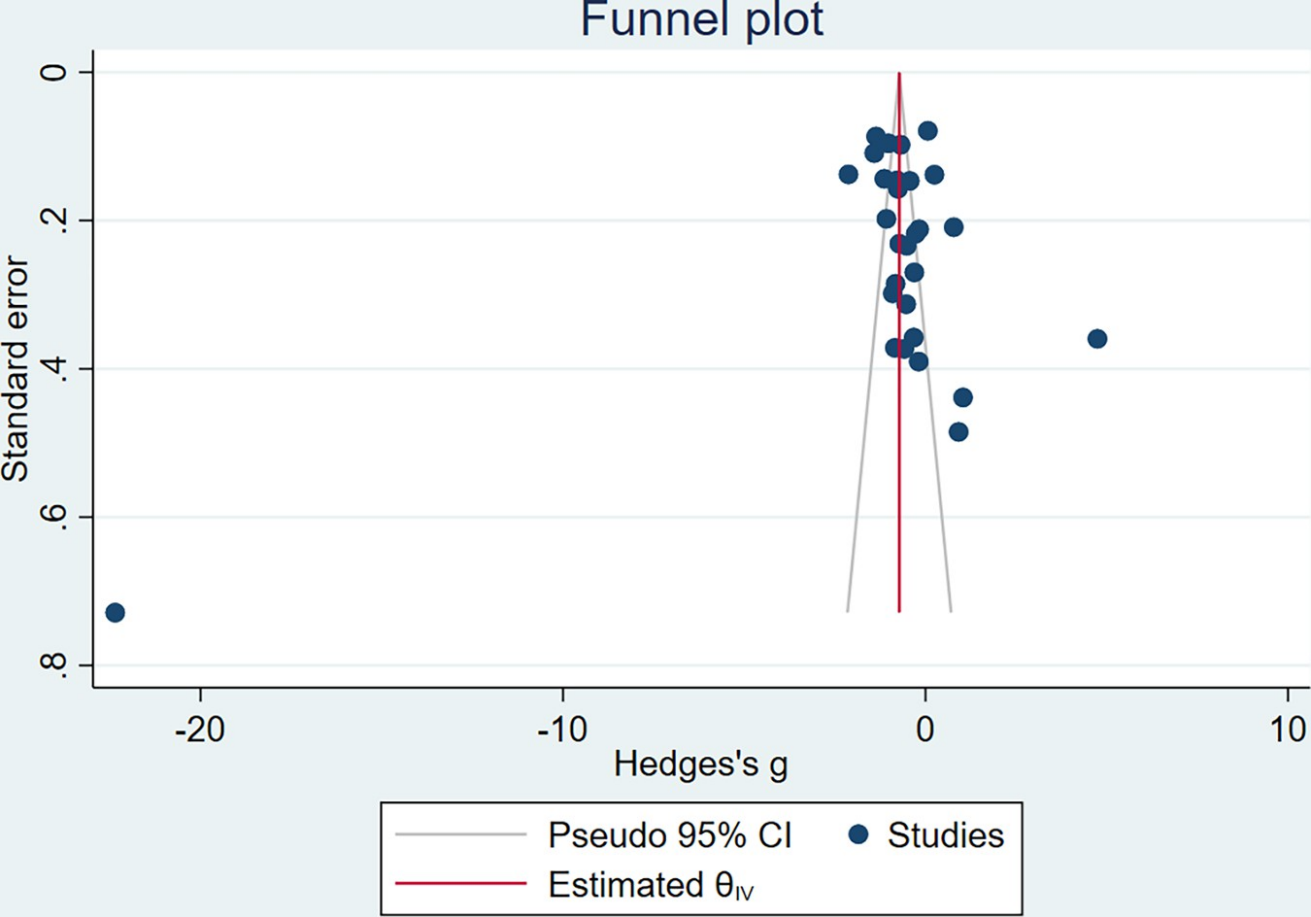
Funnel plot for meta-analysis of BDNF levels in patients with DM.

**Fig 18:**
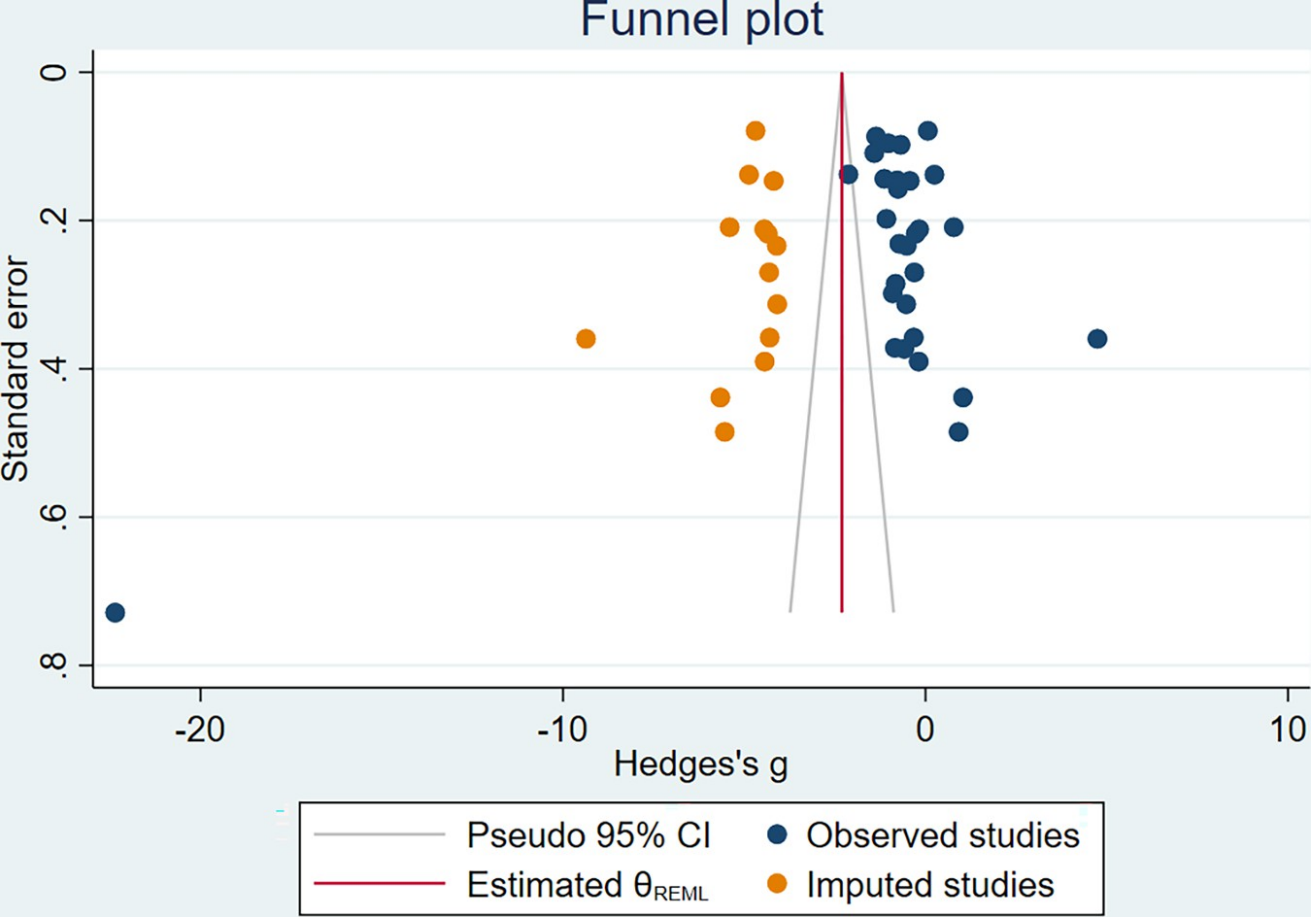
Trim and Fill funnel plot for the meta-analysis of BDNF levels in patients with DM.

Funnel plot for BDNF level was asymmetrical (Fig 17) and Eggers test revealed significant evidence of publication bias (P = 0.0024, Z = -3.04). Trim and fill method was used to adjust the effect size (Pooled estimate = -2.306 [-3.459, -1.153], z = -3.92, P<0.001, No of studies = 44) (Fig 18).

## 4. Discussion

Reportedly, this is the first study providing a meta-analysis of circulating levels of BDNF in DM patients. Although a plenty of studies have investigated BDNF levels in DM patients, the results are widely controversial. A group of studies exhibited a significantly lower level of BDNF in DM patients compared with controls [5,11–25]. However, a significant number of studies have reported unchanged [33–38] or increased BDNF levels in DM patients compared with controls [26–29]. This controversy has prompted us to investigate the circulating levels of BDNF in DM. The current study included 28 studies (23 case-control and 5 cohorts) with 2734 patients with DM and 6004 controls. The meta-analysis revealed significantly lower serum levels of BDNF in DM patients compared with controls. The meta-analysis showed lower levels of plasma BDNF in DM patients. However, the difference reached the borderline statistical significance and did not reach statistical significance. Also, DM patients with depression, DM patients with DR, as well as T2DM patients showed lower serum levels of BDNF compared with controls. Meta-regression identified disease duration as a significant moderator to explain the heterogeneity of findings in the included studies.

The association between decreased BDNF levels and CNS complications is widely investigated. Meta-analyses revealed altered BDNF levels in neurologic disorders, including Parkinson’s disease, Alzheimer’s disease, and psychiatric disorders, including autism, depression, post-traumatic stress disorder, and anxiety disorder, compared with normal controls [6,8,39–42]. On the other hand, DM is associated with neurologic and psychiatric sequelae. DM confers an increased risk for depression and anxiety disorder [43,44]. DM-mediated insulin resistance triggers beta-amyloid deposition and contributes to Alzheimer’s disease [45,46]. Also, a recent meta-analysis reported that DM is associated with an increased risk of Parkinson’s disease by 38% [47]. We postulate that the DM-mediated decrease in BDNF levels might help develop neurologic and psychiatric sequelae.

Apart from the effects of BDNF in neural regeneration and survival [48], the BDNF/TrkB/CREB signaling pathway regulates metabolism in peripheral tissues. Recent studies reveal that BDNF is associated with systemic inflammation that occurred in DM, atherosclerosis, and acute coronary syndrome, and atherosclerosis [49]. Activation of BDNF/TrkB/CREB pathway reduces hepatic gluconeogenesis, glucose levels, leptin and food intake, induces hepatic insulin signal transduction, elevates number of glycolytic fibers in the skeletal muscle and protects from pancreatic β cell loss in DM [48,50–52]. In line with these, Krabbe et al. showed that reduced BDNF levels trigger impaired glucose metabolism in T2DM patients [49]. Moreover, advanced glycation end products (AGE) are responsible for DM complications through oxidative stress, inflammation and vascular damage [53]. BDNF decreases the expression of AGE receptors and their NF-κB signaling [54]. These findings imply that decreased BDNF levels might play an important role in the pathogenesis of DM. Overall, the evidence of increased prevalence of cognitive impairment and neuropsychiatric disorders in DM patients in one hand, and the role of BDNF in mediating glucose-associated metabolic pathways in the other hand imply that DM might impact the maintenance of BDNF levels in body fluids. Also, regarding the fact that BDNF plays roles in the maintenance of CNS and metabolic functions, it can also be possible that the alterations in circulating levels of BDNF precede the occurrence of DM. In other words, whether alterations in circulating levels of BDNF are a consequence of DM or play a role in the occurrence of cognitive and metabolic dysfunctions observed in DM patients is still unclear. Further cohort studies might shed light on the exact underlying pathomechanisms responsible for alterations of BDNF levels in DM patients.

Subgroup analysis revealed that DM patients suffering from depression exhibit lower serum levels of BDNF comparing with controls. However, the effect size exceeded the overall effect size of serum BDNF levels in DM patients compared with controls. These findings are in line with evidence of BDNF levels in major depressive disorder patients [55]. A meta-analysis of circulating BDNF levels in depression revealed lower levels of BDNF in depressed patients compared with controls [6]. Interestingly, the BDNF levels correlated with depression score changes as well [6]. We postulate that the synergic effect of depression and DM in downregulation of BDNF has led to a greater decline in serum BDNF levels.

Moreover, the subgroup analysis of serum BDNF levels in DR showed that DR patients exhibit lower serum levels of BDNF compared with normal controls. Recent studies in DR animals suggest that reduced expression of BDNF in retinal glial cells and neurons might lead to neurodegeneration [56]. Although the exact mechanism of BDNF-mediated neural degeneration is still obscure, we propose several pathways to justify this phenomenon. As mentioned above, the diminished signaling of BDNF through TrkB might lead to neurodegeneration [17]. Also, reduced BDNF levels induce glutamate-mediated excitotoxic damage to postsynaptic neurons [57,58]. Last but not least, reduced BDNF levels might trigger neural apoptosis through caspase-3 activation [59].

Physical activity not only upregulates BDNF/TrkB/CREB signaling pathway but also increases insulin sensitivity BDNF is a known myokine associated with physical activity in patients with non-alcoholic fatty liver disease [60,61]. The role of physical activity as a possible moderator of circulating levels of BDNF is implicated in the literature and one of our included studies [37] investigated this correlation, however, due to a small number of studies we were not able to perform a subgroup analysis based on physical activity in our study.

**Fig 19:**
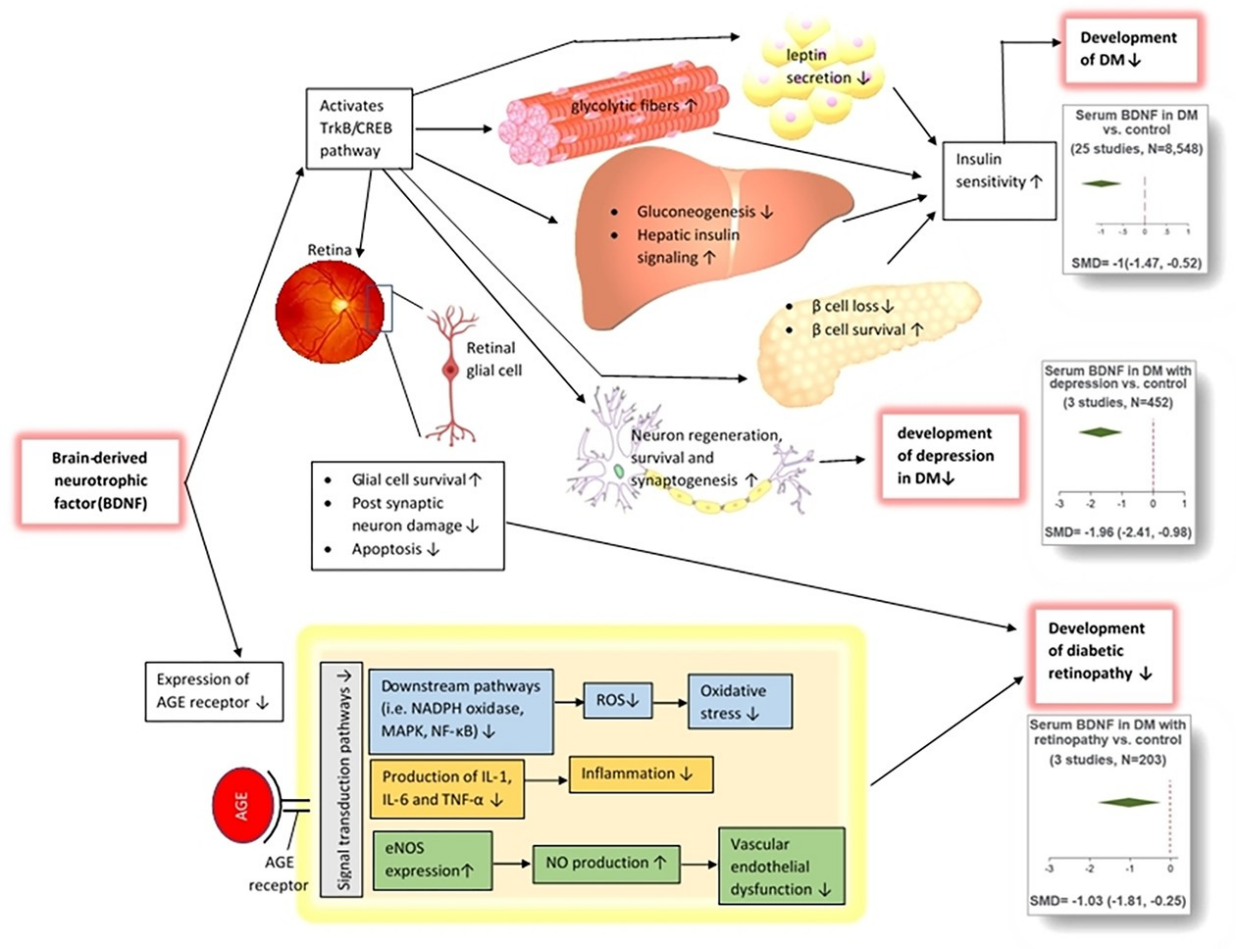
Summary of the pathophysiology and meta-analysis of the association between BDNF and DM. The image of the retina was copyright free and taken from the following link: https://www.kindpng.com/imgv/ThRohi_eye-structure-without-label-hd-png-download/. The image of the liver was copyright free and taken from the following link: https://commons.wikimedia.org/wiki/File:201405_liver.svg. The image of the skeletal muscle was adopted from the following link upon permission: https://www.123rf.com/photo_17709304_types-of-muscle-tissue-skeletal-muscle-smooth-muscle-cardiac-muscle-vector-scheme.html?vti=mpyziptnbswfclfagp-5-19. The image of the pancreas was copyright free and taken from the following link: https://app.biorender.com/illustrations/edit/60b24afd8878ed00a4f9c851. The image of the pancreas was copyright free and taken from the following link: https://togotv.dbcls.jp/en/togopic.2014.21.html. The image of the neuron was copyright free and taken from the following link: https://p.kindpng.com/picc/s/581-5810486_derived-neuron-schema-with-no-labels-neuron-psychology.png. BDNF: Brain-derived neurotrophic factor; DM: Diabetes mellitus; ROS: Reactive oxygen species; AGE: Advanced glycation endproducts; Trkb/CREB: Tyrosine kinase B/cAMP-response element binding protein; eNOS: Endothelial nitric oxide synthase; NO: Nitric oxide; NADPH: Nicotinamide adenine dinucleotide phosphate; MAPK: Mitogen-activated protein kinase; IL: Interleukin; TNF: Tumor necrosis factor.

Seemingly, we intended to perform a subgroup analysis based on the cognitive status of DM patients. However, as only two studies reported serum levels [20,51] and one study reported plasma levels [18] of BDNF in cognitively impaired DM patients, we could not perform this subgroup analysis. Summary of the pathophysiology and meta-analysis of the association between BDNF and DM is provided in Fig 19.

To investigate the effect of DM subtypes on BDNF levels, we performed a subgroup analysis on BDNF levels in T2DM patients. However, due to the lack of sufficient data on circulating levels of BDNF in T1DM and GDM, we were not able to perform a subgroup analysis based on these subtypes. Also, as BDNF is detected in the umbilical cord of pregnant women, it was postulated that umbilical cord levels of BDNF might differ in GDM patients compared with normal pregnant women. However, only two studies reported umbilical cord levels of BDNF, and thus, the number of studies did not meet the inclusion criteria for analysis [62,63]. Future experiments should address the fluctuations in BDNF levels in GDM patients, affecting both the fetus and mother.

## 5. Conclusion

Serum levels of BDNF were significantly lower among patients with DM, T2DM, DM with depression, and DM with retinopathy than the controls. Activation of the BDNF/TrkB/CREB induces hepatic insulin signal transduction, reduces hepatic gluconeogenesis, and protects from pancreatic beta-cell loss in DM. Decreased levels of BDNF lead to impaired glucose metabolism. These findings imply that decreased BDNF levels might play an important role in the pathogenesis of DM and its complications. As for future studies, we recommend addressing the association between physical activity with DM as well as subgroup analysis on patients with T1DM, GDM, and patients with cognitive complications of DM.
